# Chronic air pollution-induced subclinical airway inflammation and polygenic susceptibility

**DOI:** 10.1186/s12931-022-02179-3

**Published:** 2022-09-23

**Authors:** Sara Kress, Claudia Wigmann, Qi Zhao, Christian Herder, Michael J. Abramson, Holger Schwender, Tamara Schikowski

**Affiliations:** 1 IUF – Leibniz Research Institute for Environmental Medicine, Auf’m Hennekamp 50, 40225 Düsseldorf, Germany; 2 Medical Research School Düsseldorf, Heinrich Heine University, Düsseldorf, Germany; 3 Department of Epidemiology, School of Public Health, Cheeloo College of Medicine, Shandong University, Jinan, China; 4 Institute for Clinical Diabetology, German Diabetes Center, Leibniz Center for Diabetes Research at Heinrich Heine University Düsseldorf, Düsseldorf, Germany; 5 German Center for Diabetes Research (DZD), Partner Düsseldorf, München-Neuherberg, Germany; 6 Department of Endocrinology and Diabetology, Medical Faculty and University Hospital Düsseldorf, Heinrich Heine University Düsseldorf, Düsseldorf, Germany; 7 School of Public Health and Preventive Medicine, Monash University, Melbourne, VIC Australia; 8 Mathematical Institute, Heinrich Heine University, Düsseldorf, Germany

**Keywords:** Aged, Air pollution, Biomarkers, Gene-environment interaction, Leukotriene B4, Lung, Tumor necrosis factor-alpha

## Abstract

**Background:**

Air pollutants can activate low-grade subclinical inflammation which further impairs respiratory health. We aimed to investigate the role of polygenic susceptibility to chronic air pollution-induced subclinical airway inflammation.

**Methods:**

We used data from 296 women (69–79 years) enrolled in the population-based SALIA cohort (Study on the influence of Air pollution on Lung function, Inflammation and Aging). Biomarkers of airway inflammation were measured in induced-sputum samples at follow-up investigation in 2007–2010. Chronic air pollution exposures at residential addresses within 15 years prior to the biomarker assessments were used to estimate main environmental effects on subclinical airway inflammation. Furthermore, we calculated internally weighted polygenic risk scores based on genome-wide derived single nucleotide polymorphisms. Polygenic main and gene-environment interaction (GxE) effects were investigated by adjusted linear regression models.

**Results:**

Higher exposures to nitrogen dioxide (NO_2_), nitrogen oxides (NO_x_), particulate matter with aerodynamic diameters of ≤ 2.5 μm, ≤ 10 μm, and 2.5–10 µm significantly increased the levels of leukotriene (LT)B_4_ by 19.7% (p-value = 0.005), 20.9% (p = 0.002), 22.1% (p = 0.004), 17.4% (p = 0.004), and 23.4% (p = 0.001), respectively. We found significant effects of NO_2_ (25.9%, p = 0.008) and NO_x_ (25.9%, p-value = 0.004) on the total number of cells. No significant GxE effects were observed. The trends were mostly robust in sensitivity analyses.

**Conclusions:**

While this study confirms that higher chronic exposures to air pollution increase the risk of subclinical airway inflammation in elderly women, we could not demonstrate a significant role of polygenic susceptibility on this pathway. Further studies are required to investigate the role of polygenic susceptibility.

**Graphical Abstract:**

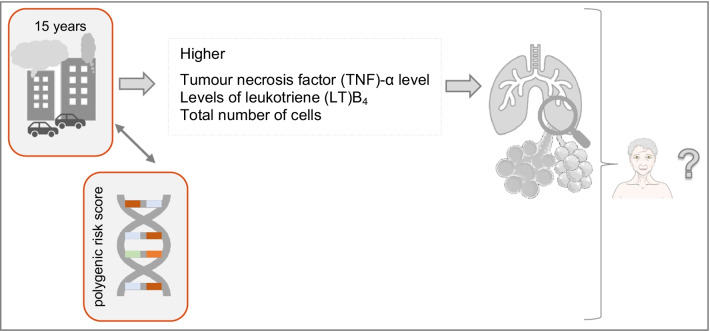

**Supplementary Information:**

The online version contains supplementary material available at 10.1186/s12931-022-02179-3.

## Background

The association between chronic air pollution exposure and respiratory health is well-established [1–3]. Regarding the underlying mechanisms, evidence supports that air pollutants can activate low-grade subclinical inflammatory biomarkers, which increase oxidative stress and further impair systemic respiratory health [3, 4].

Airway inflammatory biomarkers [5] reflect local inflammation in the lung and are directly associated with the development of respiratory morbidity [6–9]. There are various biomarkers representing different mechanisms of inflammatory pathways. Besides biomarkers of DNA oxidative damage and biomarkers of inflammation following oxidative damage (e.g. TNF-α), there are biomarkers of mediators following inflammation and oxidative damage e.g. LTB_4_ [8]. For example, alveolar macrophages ingest and clear inhaled particles and their stimulation leads to an increase of tumor necrosis factor-α (TNF-α) levels [10, 11]. LTB_4_ induces apoptosis in endothelial cells of the pulmonary artery, thereby affecting tissue injury and inflammation [12], and contributing significantly to neutrophil inflow into the airways of COPD patients [13]. Airway inflammatory biomarkers offer the possibility of detecting respiratory impacts at an early stage of disease development and could further be used to assess the individual progress of chronic obstructive pulmonary disease (COPD) [14, 15], where the elderly are considered particularly vulnerable.

In this regard, we have already reported an association between five-year means of air pollution exposure and increased levels of airway inflammatory biomarkers, such as TNF-α and leukotriene (LT) B_4_ in induced sputum from elderly individuals [16]. However, it is likely that the association between air pollution and respiratory health is influenced by individual genetic susceptibility [17–19]. Genetic variation in single nucleotide polymorphisms (SNPs) contributes to some variability in the individual response to air pollutants and the probability to develop respiratory impairment [20, 21]. SNPs acting along with environmental factors on respiratory health have already been reported in gene-environment interaction (GxE) studies [17, 18]. So far, to the best of our knowledge only one GxE study exists, which focused on SNPs belonging to the oxidative stress pathway [19]. However, many SNPs, each with a relatively small health effect, are linked within natural synergies across the entire genome. SNPs with genome-wide significance summarized in polygenic risk scores (PRS) can precisely estimate individual genetic susceptibility [22, 23].

This study aimed to investigate the role of polygenic susceptibility on the pathway of chronic air pollution exposure to subclinical airway inflammation in elderly women from the population-based longitudinal Study on the influence of Air pollution on Lung function, Inflammation and Aging (SALIA) cohort study. Adjusted linear GxE models with internally weighted PRS were fitted to the airway inflammatory biomarkers TNF-α, LTB_4_, and the total number of cells (sum of eosinophils, macrophages, neutrophils and epithelial cells) at a mean age of 74 years.

## Methods

In the ongoing SALIA cohort, 4,874 women aged 55 years living between 1985 and 1994 in the Ruhr area and the adjacent Münsterland, Germany, were enrolled. Further details of the study have been described previously [5, 24]. The study has been performed in accordance with the Declaration of Helsinki and approval was obtained from Ethics Committees of the Ruhr University, Bochum (reference number: 2732), and the Heinrich Heine University, Düsseldorf (reference number: 3507). Written informed consent from all women was received. In the current study, data from baseline, the first (2006, n = 4,027), and the second follow-up examinations (2007–10, n = 834) were analysed. The study sample was restricted to 296 women with available information on air pollution, genetics, LTB_4_ and the total number of cells at the second follow-up examination (age of 68–79 years) (292 women for TNF-α).

The airway inflammatory biomarkers (TNF-α, LTB_4_, and the total number of cells) were determined in induced sputum samples from randomly selected women. Induced sputum was collected after inhalation of vaporized isomolar saline solution for 10 min and coughing provoked according to Raulf-Heimsoth et al. [25]. Further details of the sample collection have been described previously [16]. TNF-α was measured using the PeliKine™-Tool set (CLB, Amsterdam, Netherlands) in a standard range of 1.4–1000 pg/ml and LTB_4_ by a competitive enzyme immunoassay (Assay Design, Ann Arbor, MI, USA) with a detection limit of 11.7–3000 pg/ml. The total number of cells [× 10^5^] was determined as the sum of eosinophils, macrophages, neutrophils and epithelial cells. Higher levels of airway inflammatory biomarkers represented greater subclinical inflammation and indicated higher risk for respiratory impairment. In our models, the airway inflammatory biomarker levels were log-transformed due to skewed distributions.

Individual exposures to nitrogen dioxide (NO_2_), nitrogen oxides (NO_x_), particulate matter with aerodynamic diameters of ≤ 2.5 μm, ≤ 10 μm, and 2.5–10 µm (PM_2.5_, PM_10_, PM_coarse_), and the reflectance of PM_2.5_ filters (PM_2.5 absorbance_) were estimated from average concentrations at women’s residential addresses, derived from land-use regression models assigned within the European Study of Cohorts for Air Pollution Effects (ESCAPE). Details of measurements have been described before [26, 27] and are summarised in the Additional file 1. In our study, we used the mean of annual average concentrations from baseline and first follow-up examinations with statistical centring across the participants to model chronic air pollution exposure of 15 years prior to the biomarker assessment. Higher concentrations of air pollution represented higher exposure and were standardized in interquartile ranges (IQR).

Genome-wide genotyping using the Axiom Precision Medicine Research Array GRCh37/hg19 (Affymetrix, Santa Clara, CA, USA) and quality controls [28] were performed and genetic variants were imputed against the Haplotype Reference Consortium using the Michigan Imputation Server [29] (see Additional file 1). The selection of relevant SNPs was based on the genome-wide association study (GWAS) of lung function and COPD [21] that identified 279 genome-wide significant SNPs (Additional file 2: Table S1). The biomarker-specific PRS were defined as the weighted sum of the individual number of risk alleles of the selected SNPs and calculated using the genetic risk score-interaction-training approach as recommended by Hüls et al. [30]. The data were split into a training and a test dataset. The training dataset was used to calculate the weight of each SNP on the specific biomarker and the test dataset to calculate the weighted PRS on the specific biomarker and to perform the GxE analysis. The internal weights were gained from interaction terms between each SNP and the air pollution exposure using elastic net regression models. The interaction terms were used, since there might be SNPs that were only important in areas with high air pollution exposure and these SNPs might not be included in association analyses of the main airway inflammatory effect alone. The optimal balance of sample sizes between training and test dataset in our study was determined to be 1:2 [30]. A higher PRS represented a higher number of risk alleles and was standardized in IQRs.

Descriptive study characteristics, the airway inflammatory biomarker levels, and air pollution exposure levels are presented. Linear regression models were fitted to log_e_-TNF-α level, log_e_-LTB_4_ level, and the log_e_-total number of cells with each air pollutant separately to avoid collinearity between pollutants. Using the test dataset, firstly, environmental main effects and secondly, the polygenic main effects were estimated. Furthermore, GxE effects were evaluated via a multiplicative interaction term between the air pollutant and PRS also using the test dataset. All models were adjusted for potential confounders selected a priori [19] including age, body mass index (BMI in kg/m^2^), highest education of the women or her spouse (low < 10 years, medium = 10 years, high > 10 years of education), ever-/never-smoking and second-hand smoking. Possible confounding due to residential moving could be neglected because only 1.7 percent (5 of 296) of the women changed their residential address in the last five years before the airway biomarker assessment. Results are presented in percentage changes of TNF-α level, LTB_4_ level, and the total number of cells and beta coefficients of log_e_-airway inflammatory biomarkers, as well as the corresponding 95% confidence intervals (CI) and p-values. P-values < 0.05 (two-sided) were considered as statistically significant and p-values < 0.1 were marked in the results. R version 4.1.2 [31] was used for all statistical analyses. In sensitivity analyses, first, we tested the role of binary polygenic risk (high-risk vs. low-risk group) where the group assignment was conducted according to the median of continuous polygenic risk score. Furthermore, we added indoor air pollution (exposure to mould) and heating with fossil fuels as additional potentially confounders [16]. Additionally, we investigated the main and GxE effects without adjustment for BMI as it could act more as a mediator than a confounder. We performed stratified GxE analyses to investigate potential effect modification according to chronic inflammatory respiratory conditions, such as asthma, chronic bronchitis, hay fever, cough, cough with sputum or COPD [16]. Finally, we tested another PRS including only the sentinel SNPs belonging to causal genes presented in Shrine et al. [21].

## Results

The 296 women included were on average 74.4 years old and of overweight. About a half of the participants had an education level of 10 years. Of the women, 16.6% were ever smokers and 58.5% had been exposed to second hand smoke (Table 1). The geometric means (geometric standard deviations) were 1.8 (2.1) pg/ml for TNF-α, 646.3 (2.1) pg/ml for LTB_4_, and 14.8 (2.7) × 10^5^ for the total number of cells. The median chronic air pollution exposure was lower than the annual limits of the European Union (RL 2008/50/EG) for PM_10_ (40 µg/m^3^), and NO_2_ (40 µg/m^3^), and about the same for PM_2.5_ (25 µg/m^3^) [32]. However, with regard to the annual air quality guideline levels recommended in 2021 by the World Health Organization (NO_2_: 10 µg/m^3^; PM_2.5_: 5 µg/m^3^; PM_10_: 15 µg/m^3^) [33], the median exposures were higher than recommended. The descriptive results of the SALIA study samples and the sample with air pollution and genetic assessment available, but without airway inflammatory biomarkers available indicated selection bias should be minimal (Additional file 2: Table S2).

**Table 1:** Description of the study characteristics and air pollution exposures

	SALIA study sample
N	296
Airway inflammatory biomarkers	
Arithmetic mean tumor necrosis factor-α (TNF-α) [pg/ml] (sd)	2.5 (2.4) [N = 292]
Geometric mean TNF-α [pg/ml] (gsd)	1.8 (2.1) [N = 292]
Mean log_e_-TNF-α [pg/ml] (sd)	0.6 (0.7) [N = 292]
Arithmetic mean leukotriene (LT) B_4_ [pg/ml] (sd)	867.0 (797.5)
Geometric mean LTB_4_ [pg/ml] (gsd)	646.3 (2.1)
Mean log_e_-LTB_4_ [pg/ml] (sd)	6.5 (0.8)
Arithmetic mean total number of cells [× 10^5^] (sd)	23.0 (26.3)
Geometric mean total number of cells [× 10^5^] (gsd)	14.8 (2.7)
Mean log_e_-total number of cells [× 10^5^] (sd)	2.7 (1.0)
Arithmetic mean tumor necrosis factor-α (TNF-α) [pg/ml] (sd)	867.0 (797.5)
Study characteristics	
Mean age ± sd	74.4 ± 2.7
Mean body mass index [kg/m^2^] ± sd	27.6 ± 4.6
Less than 10 years education of the participant or spouse (%)	51 (17.2)
10 years maximal education of the participant or spouse (%)	150 (50.7)
More than 10 years of the participant or spouse (%)	95 (32.1)
Ever smoker (%)	49 (16.6)
Second-hand smoking (%)	173 (58.5)
Indoor mould (%)	45 (15.2)
Heating with fossil fuels (%)	44 (15.2) [n = 289]
Chronic inflammatory respiratory conditions	81 (27.7)
Air pollution exposure	
Median chronic NO_2_ exposure [µg/m^3^] (IQR)	29.8 (10.3)
Median chronic NO_x_ exposure [µg/m^3^] (IQR)	46.5 (27.5)
Median chronic PM_2.5_ exposure [µg/m^3^] (IQR)	25.8 (2.8)
Median chronic PM_10_ exposure [µg/m^3^] (IQR)	38.8 (3.0)
Median chronic PM_coarse_ exposure [µg/m^3^] (IQR)	13.4 (2.7)
Median chronic PM_2.5 absorbance_ exposure [10^–5^/m] (IQR)	2.0 (0.6)

*sd* standard deviation, *IQR* interquartile ranges

total number of cells = the sum of eosinophils, macrophages, neutrophils and epithelial cells in induced sputum

Chronic inflammatory respiratory condition = any condition of asthma, chronic bronchitis, hay fever, cough, cough with sputum or chronic obstructive pulmonary disease

**Fig. 1:**
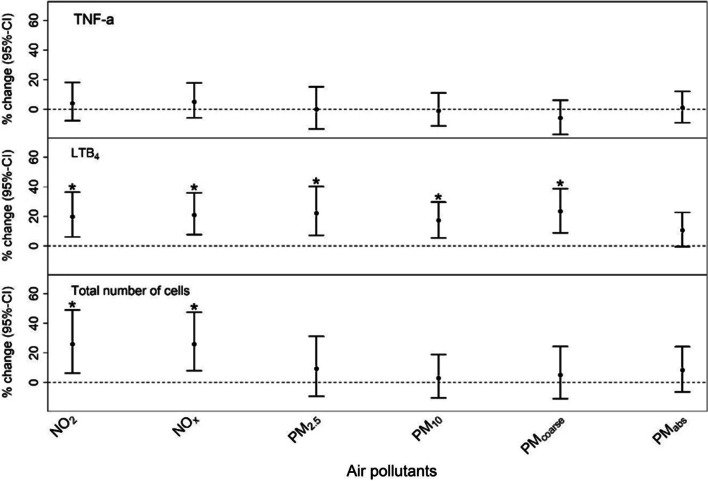
Environmental main effects of chronic air pollution exposure on airway inflammatory biomarker levels. *% change* percentage change in biomarker level, *CI* confidence interval, * = p-value: < 0.01, tumor necrosis factor-α (TNF-α), leukotriene (LT) B_4_, total number of cells = the sum of eosinophils, macrophages, neutrophils and epithelial cells, NO_2_ = nitrogen dioxide, NO_x_ = nitrogen oxides, PM_2.5/10/coarse_ = particulate matter with aerodynamic diameters ≤ 2.5/ ≤ 10/ 2.5–10 µm, PM_abs_ = PM_2.5 absorbance_, reflectance of PM_2.5_ filters Air pollutants: The mean of the annual average concentrations from baseline and first follow-up examination within a time window of 15 years prior to the airway inflammatory biomarker assessments, standardized using interquartile ranges. Adjusted for: age, body mass index (BMI in kg/m^2^), highest education of the participant or spouse, ever-/never-smoking and second-hand smoking

NO_2_ = nitrogen dioxide, NO_x_ = nitrogen oxides, PM_2.5/10/coarse_ = particulate matter with aerodynamic diameters ≤ 2.5/ ≤ 10/ 2.5–10 µm, PM_2.5 absorbance_ = reflectance of PM_2.5_ filtersThe environmental main effects are presented in Fig. 1 and together with the polygenic main effects in Additional file 2: Table S3. One IQR-increase in the exposure to NO_2_, NO_x_, PM_2.5_, PM_10_, and PM_coarse_ was significantly associated with an increase in LTB_4_ levels of 19.7% (p = 0.005), 20.9% (p = 0.002), 22.1% (p = 0.004), 17.4% (p = 0.004), and 23.3% (p = 0.001), respectively. Furthermore, we found significant effects of NO_2_ (25.9%, p = 0.008) and NO_x_ (25.9%, p = 0.004) on the total number of cells. One significant polygenic main effect was found for TNF-α using polygenic weights from the interaction terms between each SNP and NO_x_.

**Fig. 2:**
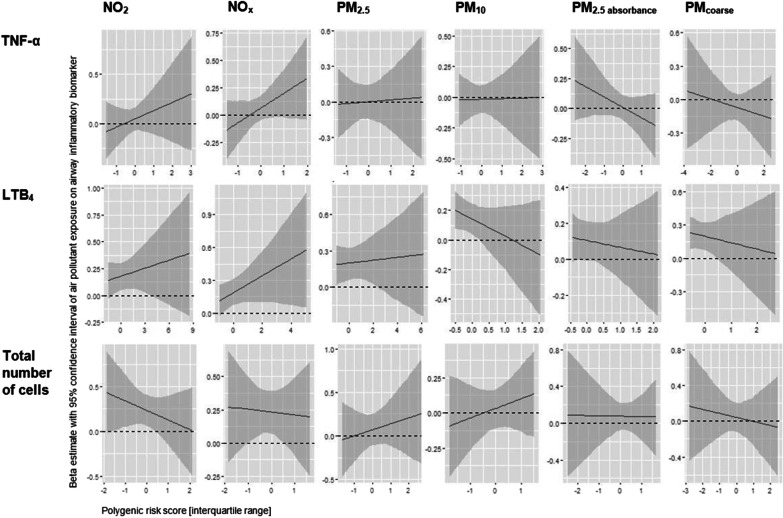
Effects of air pollutant exposure on airway inflammatory biomarkers for each IQR-increase of the PRS. log_e_-tumor necrosis factor-α (TNF-α), log_e_-leukotriene (LT) B_4_, log_e_-total number of cells = the sum of eosinophils, macrophages, neutrophils and epithelial cells, NO_2_ = nitrogen dioxide, NO_x_ = nitrogen oxides, PM_2.5/10/coarse_ = particulate matter with aerodynamic diameters ≤ 2.5/ ≤ 10/ 2.5–10 µm, PM_2.5 absorbance_ = reflectance of PM_2.5_ filters, *IQR* interquartile range, *PRS* polygenic risk score, dashed line indicates beta estimate = 0. Individual weighted polygenic risk scores (normally distributed) derived by the interaction-training approach, standardized using interquartile ranges. Air pollutants: the mean of the annual average concentrations from baseline and first follow-up examinations within a time window of 15 years prior to the airway inflammatory biomarker assessments, statistically centred across the participants, standardized using interquartile ranges. Adjusted for: age, body mass index (BMI in kg/m^2^), highest education of the participant or her spouse, ever-/never-smoking and second-hand smoking

All environmental main effects remained stable in the main GxE model. However, we did not find a significant GxE effect (Fig. 2, Additional file 2: Table S5).

In the sensitivity analyses with binary PRS (Additional file 2: Table S6), no adjustment for BMI (Additional file 2: Tables S4 and S8), and additional adjustment according to indoor air pollution (exposure to mould) and heating with fossil fuels (Additional file 2: Table S7), the trends remained stable. Excluding women with any chronic inflammatory respiratory condition did not change the GxE effects (Additional file 2: Table S9). However, this exclusion decreased the air pollution effects on LTB_4_ and increased the effects on TNF-α of NO_x_ from a 6.2 to 15.0% increase (p = 0.278 vs. 0.041) and PM_2.5 absorbance_ from a 1.0 to 16.2% increase (p = 0.884 vs. 0.037). Testing the PRS with only the sentinel SNPs belonging to causal genes included, showed consistent results and additional GxE indications for PM_2.5_ on TNF-α and PM_2.5 absorbance_ on TNF-α and the total number of cells.

## Discussion

In this study, the role of polygenic susceptibility on the pathway of chronic air pollution exposure to subclinical airway inflammation in elderly women was investigated focusing on three biomarkers TNF-α, LTB_4_, and the total number of cells in sputum. In adjusted linear regression models, we found significant environmental main effects between chronic exposures to NO_2_, NO_x_, PM_2.5_, PM_10_, and PM_coarse_ and an increase in LTB_4_ level. Furthermore, the total number of cells was significantly increased by chronic exposures to NO_2_ and NO_x_. No significant interaction effects were observed. These trends were robust in sensitivity analyses testing the dichotomized PRS, the PRS with only the sentinel SNPs belonging to causal genes included, excluding adjustment for BMI, adding potential confounders (exposure to mould and heating with fossil fuels), and the modifying effect of chronic inflammatory respiratory conditions. However, for women without any chronic inflammatory respiratory condition, a harmful environmental effect on LTB_4_ level was not confirmed.

Our results confirm that higher chronic exposures to air pollution increase the risk of subclinical airway inflammation, hence supporting the hypothesis that subclinical inflammation is an underlying mechanism of air pollution causing respiratory impairment [3, 4]. It is known that inflammation is the first response to infections, toxins and pollutants, measurable in an increase of airway inflammatory biomarker levels. However, persistent exposure to air pollutants precludes resolution of inflammation, resulting in chronically elevated levels of biomarkers of inflammation which likely cause respiratory impairment [8]. We investigated air pollution concentrations within 15 years prior to the biomarker assessment to characterise this persistent exposure. To our knowledge [8, 16], this is the first study investigating air pollution effects on airway inflammatory biomarkers in the elderly with such a long exposure window.

In regards to the environmental main effects, the high risk of NO_2_ on airway inflammatory biomarkers was also found in a study on 242 elderly COPD patients (mean age of 67.8 years) in which ESCAPE-derived exposures to PM_2.5_ and NO_2_ were examined on C-reactive protein, TNF-α, interleukin (IL)-6, IL-8, and hepatocyte growth factor. As in our study (both main analysis and analysis on women without any chronic inflammatory conditions), the association between NO_2_ and TNF-α level did not reach statistical significance, but showed a harmful trend [34]. Furthermore, there was evidence for healthy individuals as well as individuals with chronic diseases, such as asthma, COPD, and chronic bronchitis, that a doctor visit due to respiratory symptoms was associated with exposure to NO_2_ [34, 35]. While most of these study individuals were male [34, 35], our study added findings for elderly women of the harmful effects of chronic NO_x_ and NO_2_ on LTB_4_ level and the total number of cells, respectively.

Very limited studies have investigated the association between air pollution exposure and LTB_4_ level, although the role of LTB_4_ on respiratory health is well known [8, 19]. Our study helps to clarify the evidence and supports the importance of LTB_4_ through showing significant harmful effects of exposure to NO_2_, NO_x_, PM_2.5_, PM_10_, and PM_coarse_. The decreasing trend of air pollution effects on LTB_4_ level in women without any chronic inflammatory respiratory condition could indicate the importance for elderly women with COPD. However, the results must be taken with caution because of the small sample sizes.

Additionally, there are some studies of the association between genetics and airway inflammatory biomarkers. In the family-based Framingham Heart Study, heritability and candidate gene associations of inflammatory biomarkers (overlap with the biomarkers included here: TNF-α) were investigated, but no correlations with TNF-α were found [36]. Moreover, in a GWAS of systemic inflammatory biomarkers of subjects with COPD [37] and a meta-analysis of current and former smokers with or without COPD [38], none of the SNPs was associated with TNF-α level (overlap: TNF-α). In a meta-analysis of 34 studies involving 5,477 asthma patients and 5,962 controls TNF-α rs1800629 (no overlap) polymorphism was only significantly associated with asthma risk in Asian populations, but not in Caucasian populations [39]. In another meta-analysis, TNF-α -308 G/A polymorphisms (no overlap) was also associated only among Asian populations with an increased risk for COPD, but not in non-Asian individuals [40]. There are fewer studies considering genetic effects on LTB_4_ and the total number of cells. Regarding LTB_4_, no significant association was found with LTA4H regulatory variant rs2660845 (no overlap) in European late-onset asthma individuals [41]. Our study is consistent with these results by showing no stable significant polygenic effects, although these studies are only comparable with our study to a limited extent due to the different genetic approaches and the rarely examined elderly population.

The association between chronic air pollution and respiratory health on airway inflammation is likely to be influenced by individual genetic susceptibility [17, 18, 20]. To the best of our knowledge, there is only one GxE study of air pollution-induced airway inflammation. This study focused on SNPs relevant to the oxidative stress pathway [19], making it the first study considering SNPs in natural synergies across the entire genome, summarized in PRS [22, 23]. While Hüls et al. [19] found the strongest GxE for LTB_4_, we found no GxE effects. Possible explanations are, on one hand, that the GWAS of lung function and COPD [21] used in our study did not cover the relevant SNPs for subclinical airway inflammation [42], and especially not those SNPs that interacted with air pollution. SNPs that were only important in areas with low air pollution might not have reached genome-wide significance in the GWAS, but would be identified in genome-wide association interaction studies and genome-wide by environmental interaction studies [43].

On the other hand, GxE effects could have a different starting point within the pathway. With our approach, we examined GxE effects on subclinical airway inflammation, where the GxE effect could also influence systemic inflammation instead of subclinical inflammation. Maybe the subclinical inflammation induced by air pollution was not dependent on polygenic susceptibility, but the polygenic susceptibility affected the development of subclinical inflammation towards either respiratory impairment or recovery. Hüls et al. [44] have already shown that an air pollution-associated improvement of lung function depends on the individual genetic risk, which might be associated with subclinical airway inflammation. This would support our hypothesis. However, further studies examining the whole pathway are necessary to confirm this. Our findings are generalizable to other healthy elderly Caucasian women. With regard to the different genetic effects between Asian and Caucasian populations in other studies, it is likely that polygenic susceptibility as well as the GxE effects differ between ancestry groups.

Strengths of our study are the population-based design, standardized procedures to measure the biomarkers, and standardized and comparable ESCAPE air pollution data covering a time window of 15 years prior to the biomarker assessments. Several sensitivity analyses were performed to confirm our findings, rule out selection bias, and investigate modifying effects by chronic inflammatory respiratory conditions.

However, there were also some limitations. In the SALIA cohort, the effect of air pollution and the GxE effect could be underestimated due to the loss to follow-up examination of women with less education, higher air pollution exposure and worse respiratory health [19]. In our study, TNF-α results must be treated with caution because about 40% of the measurements were below the detection limit [19]. Due to the lack of genome-wide interaction studies and GWAS on specific airway inflammation biomarkers, the polygenic susceptibility to subclinical airway inflammation was based on SNP selection regarding general lung function and COPD, which might result in different SNPs and could consequently affect polygenic main or GxE effects. Using external weights would improve the external validity of GxE results. In addition, a lack of statistical power due to the small sample sizes could be a reason for not identifying interaction effects.

## Conclusions

While this study confirmed that higher chronic exposure to air pollution increased the risk of subclinical airway inflammation in elderly women, we could not detect a significant role of polygenic susceptibility on this pathway. Our study added to the evidence on airway inflammation in elderly women, especially for the harmful effects of NO_2_ and NO_x_ on subclinical inflammation, considering a 15-year long exposure window, and the harmful air pollution effects on LTB_4_ levels. Further GxE studies including genome-wide derived SNPs are required to investigate the role of polygenic susceptibility to air pollution-induced subclinical airway inflammation and to provide further insights into underlying mechanisms.

## Take home message

Higher chronic exposure to air pollution increases the risk of subclinical airway inflammation in elderly women. Our study could not detect a significant role of polygenic susceptibility in air pollution-induced subclinical airway inflammation and further studies are required.

## Supplementary Information

**Additional file 1.** Air pollution assignment within the European Study of Cohorts for Air Pollution Effects. Details of air pollution measurements. Genotyping, quality control and imputation. Details of genotyping, quality control and imputation.

**Additional file 2: Table S1.** Information on 279 Single Nucleotide Polymorphisms (SNPs) from the genome-wide association study on lung function and chronic obstructive lung disease by Shrine et al. (2019; n = 400,102). 278 SNPs were included in our calculation of the polygenic risk score. Marked SNPs (*) were also included in our polygenic risk score of sentinel SNPs belonging to causal genes (see Additional file 2: Table S10). Annotation data of the 278 SNPs included in our calculation of the polygenic risk score such as *CHROM* chromosome, *rsID* reference SNP cluster ID, *POS* reference position, *REF* reference allele, *ALT* alternative non-reference allele, *SNP* CHROM:POS:REF:ALT, *MAF* minor allele frequency in the specific cohort as the second most common allele count from the number of alleles in called genotypes in the specific cohort, *TYPED* indicates if the variant was genotyped or imputed, *R2* imputation quality as the estimated value of the squared correlation between imputed genotypes and true/unobserved genotypes, *ER2* empirical R2 for genotyped variants (not calculated for imputed variants), * = SNPs included in our polygenic risk score of sentinel SNPs belonging to causal genes. **Table S2.** Descriptive statistics on each study and model sample, airway inflammatory biomarker levels and air pollution exposures in the SALIA cohort. Descriptive statistics on each study and model sample using arithmetic and geometric mean and standard deviation of airway inflammatory biomarkers (tumor necrosis factor-α, leukotriene B4, and the sum of eosinophils, macrophages, neutrophils and epithelial cells in induced sputum), study characteristics including mean age, body mass index, education, smoking, indoor air pollution, and chronic inflammatory respiratory condition defined as any condition of asthma, chronic bronchitis, hay fever, cough, cough with sputum or chronic obstructive pulmonary disease, and median and interquartile ranges of chronic air pollution exposure of nitrogen dioxide, nitrogen oxides, particulate matter with aerodynamic diameters ≤ 2.5/ ≤ 10/2.5–10 µm, reflectance of PM_2.5_ filters calculated as the mean of the annual average concentrations from baseline and first follow-up examinations within a time window of 15 years before the biomarker assessments, statistically centred across the participants. **Table S3.** Environmental main effects of chronic air pollution exposure and the polygenic main effects on natural log-transformed airway inflammatory biomarker level in elderly women using adjusted linear regression models in test dataset. Environmental main effects as the effect of chronic air pollution exposure (nitrogen dioxide, nitrogen oxides, particulate matter with aerodynamic diameters of ≤ 2.5/ ≤ 10/2.5–10 µm, reflectance of PM_2.5_ filters calculated as the mean of the annual average concentrations from baseline and first follow-up examinations within a time window of 15 years prior to the biomarker assessments, statistically centred across the participants, standardized using interquartile ranges) on airway inflammatory biomarkers (tumor necrosis factor-α, leukotriene B4, and the sum of eosinophils, macrophages, neutrophils and epithelial cells in induced sputum) adjusted for: age, body mass index (BMI in kg/m^2^), highest education of the participant or her spouse (low < 10 years, medium = 10 years, high > 10 years of education), ever-/never-smoking, and second-hand smoking using adjusted linear regression models in test dataset: beta estimate with 95% confidence intervals on natural log-transformed airway inflammatory biomarker level and percentage change with 95% confidence interval in airway inflammatory biomarker level. P-values < 0.05 are highlighted bold and p-values < 0.1 cursive. Polygenic main effects as the effect of polygenic risk score (normally distributed (Shapiro–Wilk normality test: n = 194, p-value = 0.002) on airway inflammatory biomarkers (tumor necrosis factor-α, leukotriene B4, and the sum of eosinophils, macrophages, neutrophils and epithelial cells in induced sputum) adjusted for: age, body mass index (BMI in kg/m^2^), highest education of the participant or her spouse (low < 10 years, medium = 10 years, high > 10 years of education), ever-/never-smoking, and second-hand smoking using adjusted linear regression models in test dataset: beta estimate with 95% confidence intervals on natural log-transformed airway inflammatory biomarker level and percentage change with 95% confidence interval in airway inflammatory biomarker level. P-values < 0.05 are highlighted bold and p-values < 0.1 cursive. The polygenic weights are gained from the interaction terms between each SNP and the air pollution exposure using elastic net regression models, hence it results one polygenic main effect with each air pollutant per airway inflammatory biomarker. **Table S4.** The environmental main effects of chronic air pollution exposure and the polygenic main effects on natural log-transformed airway inflammatory biomarker level in elderly women using adjusted linear regression models (without adjustment for body mass index) in test dataset. Environmental main effects as the effect of chronic air pollution exposure (nitrogen dioxide, nitrogen oxides, particulate matter with aerodynamic diameters of ≤ 2.5/ ≤ 10/2.5–10 µm, reflectance of PM_2.5_ filters calculated as the mean of the annual average concentrations from baseline and first follow-up examinations within a time window of 15 years prior to the biomarker assessments, statistically centred across the participants, standardized using interquartile ranges) on airway inflammatory biomarkers (tumor necrosis factor-α, leukotriene B4, and the sum of eosinophils, macrophages, neutrophils and epithelial cells in induced sputum) adjusted for: age, highest education of the participant or her spouse (low < 10 years, medium = 10 years, high > 10 years of education), ever-/never-smoking, and second-hand smoking using adjusted linear regression models in test dataset: beta estimate with 95% confidence intervals on natural log-transformed airway inflammatory biomarker level and percentage change with 95% confidence interval in airway inflammatory biomarker level. P-values < 0.05 are highlighted bold and p-values < 0.1 cursive. Polygenic main effects as the effect of polygenic risk score (normally distributed (Shapiro–Wilk normality test: n = 194, p-value = 0.002) on airway inflammatory biomarkers (tumor necrosis factor-α, leukotriene B4, and the sum of eosinophils, macrophages, neutrophils and epithelial cells in induced sputum) adjusted for: age, highest education of the participant or her spouse (low < 10 years, medium = 10 years, high > 10 years of education), ever-/never-smoking, and second-hand smoking using adjusted linear regression models in test dataset: beta estimate with 95% confidence intervals on natural log-transformed airway inflammatory biomarker level and percentage change with 95% confidence interval in airway inflammatory biomarker level. P-values < 0.05 are highlighted bold and p-values < 0.1 cursive. The polygenic weights are gained from the interaction terms between each SNP and the air pollution exposure using elastic net regression models, hence it results one polygenic main effect with each air pollutant per airway inflammatory biomarker. **Table S5.** Gene-environment interaction effects between the weighted polygenic risk score and chronic air pollution exposure on natural log-transformed airway inflammatory biomarker levels in elderly women using adjusted linear regression models in test dataset. Gene-environment interaction effects between the weighted polygenic risk score (derived by the interaction-training approach, standardized using interquartile ranges) and chronic air pollution exposure (nitrogen dioxide, nitrogen oxides, particulate matter with aerodynamic diameters of ≤ 2.5/ ≤ 10/2.5–10 µm, reflectance of PM_2.5_ filters calculated as the mean of the annual average concentrations from baseline and first follow-up examinations within a time window of 15 years prior to the biomarker assessments, statistically centred across the participants, standardized using interquartile ranges) on natural log-transformed airway inflammatory biomarker levels (tumor necrosis factor-α, leukotriene B4, and the sum of eosinophils, macrophages, neutrophils and epithelial cells in induced sputum) adjusted for: age, body mass index (BMI in kg/m^2^), highest education of the participant or her spouse (low < 10 years, medium = 10 years, high > 10 years of education), ever-/never-smoking, and second-hand smoking using adjusted linear regression models in test dataset: beta estimate with 95% confidence intervals on natural log-transformed airway inflammatory biomarker level and percentage change with 95% confidence interval in airway inflammatory biomarker level. P-values < 0.05 are highlighted bold and p-values < 0.1 cursive. **Table S6.** Gene-environment interaction effects between the weighted binary polygenic risk score (genetic low-risk vs. high-risk group) and chronic air pollution exposure on natural log-transformed airway inflammatory biomarker levels in elderly women using adjusted linear regression models in test dataset. Gene-environment interaction effects between the weighted binary polygenic risk score (derived by the interaction-training approach, dichotomized using the median of weighted polygenic risk score) and chronic air pollution exposure (nitrogen dioxide, nitrogen oxides, particulate matter with aerodynamic diameters of ≤ 2.5/ ≤ 10/2.5–10 µm, reflectance of PM_2.5_ filters calculated as the mean of the annual average concentrations from baseline and first follow-up examinations within a time window of 15 years prior to the biomarker assessments, statistically centred across the participants, standardized using interquartile ranges) on natural log-transformed airway inflammatory biomarker levels (tumor necrosis factor-α, leukotriene B4, and the sum of eosinophils, macrophages, neutrophils and epithelial cells in induced sputum) adjusted for: age, body mass index (BMI in kg/m^2^), highest education of the participant or her spouse (low < 10 years, medium = 10 years, high > 10 years of education), ever-/never-smoking, and second-hand smoking using adjusted linear regression models in test dataset: beta estimate with 95% confidence intervals on natural log-transformed airway inflammatory biomarker level and percentage change with 95% confidence interval in airway inflammatory biomarker level. P-values < 0.05 are highlighted bold and p-values < 0.1 cursive. **Table S7.** Gene-environment interaction effects between the weighted polygenic risk score and chronic air pollution exposure on natural log-transformed airway inflammatory biomarker levels in elderly women using linear regression models in test dataset with additional adjustment according to indoor air pollution (exposure to mould), and heating with fossil fuels. Gene-environment interaction effects between the weighted polygenic risk score (derived by the interaction-training approach, standardized using interquartile ranges) and chronic air pollution exposure (nitrogen dioxide, nitrogen oxides, particulate matter with aerodynamic diameters of ≤ 2.5/ ≤ 10/2.5–10 µm, reflectance of PM_2.5_ filters calculated as the mean of the annual average concentrations from baseline and first follow-up examinations within a time window of 15 years prior to the biomarker assessments, statistically centred across the participants, standardized using interquartile ranges) on natural log-transformed airway inflammatory biomarker levels (tumor necrosis factor-α, leukotriene B4, and the sum of eosinophils, macrophages, neutrophils and epithelial cells in induced sputum) adjusted for: age, body mass index (BMI in kg/m^2^), highest education of the participant or her spouse (low < 10 years, medium = 10 years, high > 10 years of education), ever-/never-smoking, second-hand smoking, indoor air pollution (exposure to mould), and heating with fossil fuels using adjusted linear regression models in test dataset: beta estimate with 95% confidence intervals on natural log-transformed airway inflammatory biomarker level and percentage change with 95% confidence interval in airway inflammatory biomarker level. P-values < 0.05 are highlighted bold and p-values < 0.1 cursive. **Table S8.** Gene-environment interaction effects between the weighted polygenic risk score and chronic air pollution exposure on natural log-transformed airway inflammatory biomarker levels in elderly women using linear regression models in test dataset with no adjustment according to body mass index. Gene-environment interaction effects between the weighted polygenic risk score (derived by the interaction-training approach, standardized using interquartile ranges) and chronic air pollution exposure (nitrogen dioxide, nitrogen oxides, particulate matter with aerodynamic diameters of ≤ 2.5/ ≤ 10/2.5–10 µm, reflectance of PM_2.5_ filters calculated as the mean of the annual average concentrations from baseline and first follow-up examinations within a time window of 15 years prior to the biomarker assessments, statistically centred across the participants, standardized using interquartile ranges) on natural log-transformed airway inflammatory biomarker levels (tumor necrosis factor-α, leukotriene B4, and the sum of eosinophils, macrophages, neutrophils and epithelial cells in induced sputum) adjusted for: age, highest education of the participant or her spouse (low < 10 years, medium = 10 years, high > 10 years of education), ever-/never-smoking, and second-hand smoking using adjusted linear regression models in test dataset: beta estimate with 95% confidence intervals on natural log-transformed airway inflammatory biomarker level and percentage change with 95% confidence interval in airway inflammatory biomarker level. P-values < 0.05 are highlighted bold and p-values < 0.1 cursive. **Table S9.** Gene-environment interaction effects between the weighted polygenic risk score and chronic air pollution exposure on natural log-transformed airway inflammatory biomarker levels in elderly women using adjusted linear regression models in test dataset excluding women with any chronic inflammatory respiratory condition. Gene-environment interaction effects between the weighted polygenic risk score (derived by the interaction-training approach, standardized using interquartile ranges) and chronic air pollution exposure (nitrogen dioxide, nitrogen oxides, particulate matter with aerodynamic diameters of ≤ 2.5/ ≤ 10/2.5–10 µm, reflectance of PM_2.5_ filters calculated as the mean of the annual average concentrations from baseline and first follow-up examinations within a time window of 15 years prior to the biomarker assessments, statistically centred across the participants, standardized using interquartile ranges) on natural log-transformed airway inflammatory biomarker levels (tumor necrosis factor-α, leukotriene B4, and the sum of eosinophils, macrophages, neutrophils and epithelial cells in induced sputum) adjusted for: age, body mass index (BMI in kg/m^2^), highest education of the participant or her spouse (low < 10 years, medium = 10 years, high > 10 years of education), ever-/never-smoking, and second-hand smoking in women without any condition of asthma, chronic bronchitis, hay fever, cough, cough with sputum or chronic obstructive pulmonary disease using adjusted linear regression models in test dataset: beta estimate with 95% confidence intervals on natural log-transformed airway inflammatory biomarker level and percentage change with 95% confidence interval in airway inflammatory biomarker level. P-values < 0.05 are highlighted bold and p-values < 0.1 cursive. **Table S10.** Gene-environment interaction effects between the weighted polygenic risk score including only the Sentinel SNPs belonging to causal genes and chronic air pollution exposure on natural log-transformed airway inflammatory biomarker levels in elderly women using adjusted linear regression models in test dataset. Gene-environment interaction effects between the weighted polygenic risk score (derived by the interaction-training approach, standardized using interquartile ranges, including only the Sentinel SNPs belonging to causal genes) and chronic air pollution exposure (nitrogen dioxide, nitrogen oxides, particulate matter with aerodynamic diameters of ≤ 2.5/ ≤ 10/ 2.5–10 µm, reflectance of PM_2.5_ filters calculated as the mean of the annual average concentrations from baseline and first follow-up examinations within a time window of 15 years prior to the biomarker assessments, statistically centred across the participants, standardized using interquartile ranges) on natural log-transformed airway inflammatory biomarker levels (tumor necrosis factor-α, leukotriene B4, and the sum of eosinophils, macrophages, neutrophils and epithelial cells in induced sputum) adjusted for: age, body mass index (BMI in kg/m^2^), highest education of the participant or her spouse (low < 10 years, medium = 10 years, high > 10 years of education), ever-/never-smoking, and second-hand smoking using adjusted linear regression models in test dataset: beta estimate with 95% confidence intervals on natural log-transformed airway inflammatory biomarker level and percentage change with 95% confidence interval in airway inflammatory biomarker level. P-values < 0.05 are highlighted bold and p-values < 0.1 cursive.

## Data Availability

The data set of the SALIA cohort study is not publicly available due to the data protection and privacy laws in the European Union but are available from the corresponding author on reasonable request.

## Abbreviations

BMI: Body mass index
CI: 95% Confidence intervals
COPD: Chronic obstructive pulmonary disease
ESCAPE: European Study of Cohorts for Air Pollution Effects
GWAS: Genome-wide association study
GxE: Gene-environment interaction
IL: Interleukin
IQR: Interquartile range
LTB_4_: Leukotriene B_4_
NO_2_: Nitrogen dioxide
NO_x_: Nitrogen oxides
PM_2.5_: Particulate matter with aerodynamic diameters of ≤ 2.5 μm
PM_2.5 absorbance_: The reflectance of PM_2.5_ filters
PM_10_: Particulate matter with aerodynamic diameters of ≤ 10 μm
PM_coarse_: Particulate matter with aerodynamic diameters of 2.5–10 µm
PRS: Polygenic risk scores
SALIA: Study on the influence of Air pollution on Lung function, Inflammation and Aging
SD: Standard deviation
SNP: Single nucleotide polymorphisms
TNF-α: Tumor necrosis factor-α
