# Supplementary material for: Chronic air pollution-induced subclinical airway inflammation and polygenic susceptibility
Source: Respir Res. 2022 Sep 23;23:265. doi: 10.1186/s12931-022-02179-3 (PMC9508765; doi:10.1186/s12931-022-02179-3)
Supplement: Supplementary file 2 — Additional file 2: Table S1. Information on 279 Single Nucleotide Polymorphisms (SNPs) from the genome-wide association study on lung function and chronic obstructive lung disease by Shrine et al. (2019; n = 400,102). 278 SNPs were included in our calculation of the polygenic risk score. Marked SNPs (*) were also included in our polygenic risk score of sentinel SNPs belonging to causal genes (see Additional file 2: Table S10). Annotation data of the 278 SNPs included in our calculation of the polygenic risk score such as CHROM chromosome, rsID reference SNP cluster ID, POS reference position, REF reference allele, ALT alternative non-reference allele, SNP CHROM:POS:REF:ALT, MAF minor allele frequency in the specific cohort as the second most common allele count from the number of alleles in called genotypes in the specific cohort, TYPED indicates if the variant was genotyped or imputed, R2 imputation quality as the estimated value of the squared correlation between imputed genotypes and true/unobserved genotypes, ER2 empirical R2 for genotyped variants (not calculated for imputed variants), * = SNPs included in our polygenic risk score of sentinel SNPs belonging to causal genes. Table S2. Descriptive statistics on each study and model sample, airway inflammatory biomarker levels and air pollution exposures in the SALIA cohort. Descriptive statistics on each study and model sample using arithmetic and geometric mean and standard deviation of airway inflammatory biomarkers (tumor necrosis factor-α, leukotriene B4, and the sum of eosinophils, macrophages, neutrophils and epithelial cells in induced sputum), study characteristics including mean age, body mass index, education, smoking, indoor air pollution, and chronic inflammatory respiratory condition defined as any condition of asthma, chronic bronchitis, hay fever, cough, cough with sputum or chronic obstructive pulmonary disease, and median and interquartile ranges of chronic air pollution exposure of nitrogen diox [file 12931_2022_2179_MOESM2_ESM.docx]

**Additional file 2**

Chronic air pollution-induced subclinical airway inflammation and polygenic susceptibility

Sara Kress, Claudia Wigmann, Qi Zhao, Christian Herder, Michael Abramson, Holger Schwender, Tamara Schikowski

# Text details

## Air pollution assignment within the European Study of Cohorts for Air Pollution Effects

Air pollution exposures were assigned within the European Study of Cohorts for Air Pollution Effects (ESCAPE) [1,2]. ESCAPE conducted 14-day measurements of PM_2.5/ 10/ 2.5 absorbance_ in each cold, warm and intermediate temperature season between October 2008 and November 2009 from 20 monitoring sites. NO_2_ and NO_x_ were monitored at 40 sites. The concentration of PM_coarse_ was calculated by subtracting PM_2.5_ from PM_10_. With a central reference monitoring site, which measured concentrations of air pollution using the same instruments continuously for a complete year, the values were adjusted for the true long-term average of the observation period. Finally, land-use regression models predicted the air pollution concentrations at the home addresses for each participant at each follow-up examination respectively. The data quality was examined and assured, e.g. only sites with high-quality data for at least 75% of the days in a year were used.

## Genotyping, quality control and imputation

Genome-wide genotyping was performed in December 2016/ January 2017 in 468 blood and saliva samples and additional in November 2020 in 284 blood and saliva samples using the Axiom Precision Medicine Research Array (Affymetrix, Santa Clara, CA, USA) (GRCh37/hg19) resulting in 871,262 variants.

In the pre-imputation quality control [3] variants on chromosome 0, insert/deletion variants, variants with low minor allele frequency (<0.01), and low call rates (<0.95) were excluded. After that, duplicated individuals, individuals with sex-mismatch, with low call rates (<0.95), with minimal heterozygosity (inbreeding coefficient 0.1), highly related individuals (identity-by-descent analysis with ld.tresh=0.2 and kin.tresh=0.1), as well as individuals belonging to non-European ancestry group (Tukey's rule based on the 1-10 eigenvectors from Principal Component Analysis), and violations of Hardy-Weinberg (p<10^-6^) were removed. Finally, SNPs that deviate from Hardy-Weinberg equilibrium (p<10^-6^) were removed. Strand designation/ strand flips correction were done. 586 individuals and 410,652 SNPs passed the pre-imputation quality control

To find haplotype segments that are shared by study individuals and the HRC r1.1 2016 (GRCh37/hg19), we did a genotype imputation with minimac4 1.5.7 using the Michigan Imputation Server [4]. 386,710 SNPs passed the quality control of the Michigan Imputation Server.

In post-imputation processing multi-allelic markers and variants with low minor allele frequency (<0.01), as well as variants with low imputation quality (R²<0.3) were excluded. The respective rs-ids were merged by chromosome, position, reference allele, and alternative allele using HRC.r1-1.GRCh37.wgs.mac5.sites.vcf.gz [4]. After post-imputation quality control 586 individuals and 7,643,653 SNPs remained.

References

1. Beelen R, Hoek G, Vienneau D, et al. Development of NO2 and NOx land use regression models for estimating air pollution exposure in 36 study areas in Europe – The ESCAPE project. Atmospheric Environment 2013;72:10–23. DOI:10.1016/j.atmosenv.2013.02.037.

2. Eeftens M, Beelen R, Hoogh K de, et al. Development of Land Use Regression models for PM(2.5), PM(2.5) absorbance, PM(10) and PM(coarse) in 20 European study areas; results of the ESCAPE project. Environmental science & technology 2012;46;20:11195–205. DOI:10.1021/es301948k.

3. Reed E, Nunez S, Kulp D, et al. A guide to genome-wide association analysis and post-analytic interrogation. Statistics in medicine 2015;34;28:3769–92. DOI:10.1002/sim.6605.

4. Das S, Forer L, Schönherr S, et al. Next-generation genotype imputation service and methods. Nature genetics 2016;48;10:1284–7. DOI:10.1038/ng.3656.
